# Invited Editorial: Despite COVID-19, Influenza Must Not Be Relegated to “Only the Sniffles”

**DOI:** 10.3390/vaccines8030445

**Published:** 2020-08-07

**Authors:** Graham Pawelec

**Affiliations:** 1 Department of Immunology, University of Tübingen, 72072 Tübingen, Germany; graham.pawelec@uni-tuebingen.de or grahampawelec@cantab.net; 2 Health Sciences North Research Institute, Sudbury, ON P3E 2H2, Canada

As the current COVID-19 pandemic continues to rage worldwide, it has emerged that the 2019–2020 influenza season has been milder and shorter than usual in the northern hemisphere, presumably due to enforced social distancing. Thus, for example, in countries relatively successful in containing SARS-CoV-2, influenza burden and pneumonia deaths were significantly lower than expected for the season [1]. Nonetheless, seasonal influenza remains a major public health concern and despite the fact that vaccines have been available for decades, they are not optimally protective for all segments of the population [2]. Only a small fraction of the vast resources freed up for research into developing a SARS-CoV-2 vaccine has ever been invested in influenza vaccines, and there remains an urgent need for improvements to prevent the average 500,000 influenza deaths per year usually recorded. Five recent reviews published in this journal have addressed different aspects of this challenge, from improvements to conventional seasonal vaccines and the use of RNA as the immunogen, the development of a universal vaccine applicable for every influenza season, the necessity for vaccines to stimulate cellular as well as humoral immunity, and importantly, how to measure the effectiveness of vaccines in development and practice, and the impact of previous exposures on a new challenge. Together, they form a blueprint for efforts to improve the efficiency of influenza vaccination to mitigate the acute and chronic effects of an infectious disease that remains a major public health challenge even during the COVID-19 pandemic.

As Harding and Heaton note [3], because influenza viruses mutate rapidly, prophylactic vaccines must be updated every year according to estimates of which strains will be circulating. Clearly, the development of a universal vaccine offering broadly protective immunity against multiple strains would be far preferable, but despite much effort these are not yet available, and there are no guarantees that they will be possible. Hence, Harding and Heaton argue that it remains necessary to evolve strategies for improving current standard seasonal vaccines. They review the many diverse techniques and technologies that are being investigated in an effort to improve seasonal vaccines. One has to say that there is certainly a vast amount of room for improvement. In fact, were the current procedure and infrastructure not already so firmly in place, nobody in their right mind would ever develop such a method nowadays—a method that has remained essentially unchanged for half a century. First, huge effort is required to predict the next season’s predominant influenza strains, which is more of an educated guess, and it is not always correct. Next, for almost all vaccines currently manufactured, virus candidates must be selected and adapted to growth in hens’ eggs. There has been a great deal of discussion about the pros and cons of this antediluvian technique, as summarized in the paper. The use of cell culture systems may overcome some of these difficulties, but it still requires virus adaptation and comes with many other provisos. More recently, recombinant genetic engineering techniques are being introduced, among which baculovirus production has been FDA approved. Finally, formulations including adjuvants, some FDA-approved, are beginning to come on-line. Harding and Heaton then go on to discuss several of the more exciting new approaches under development, many of which originated from investigations by cancer vaccine developers and which are currently greatly amplified by efforts to produce SARS-CoV-2 vaccines.

Of these new approaches, the use of RNA vaccines is perhaps one of the most likely to yield breakthrough results, as reviewed in the paper by Scorza and Pardi [4]. Much work has been done on developing stabilized forms of RNA for use in vaccines, which can also be self-adjuvanting and do not necessarily need a cold chain for delivery. The latter consideration is not so important in industrialized countries but can be crucial in low- and middle-income countries. Several RNA vaccines have been tested in preclinical models, mostly mouse, but also in those closer to humans, i.e., ferrets and pigs. The pros and cons of self-amplifying influenza virus RNA vaccines, non-replicating influenza virus mRNA vaccines, and industrial development strategies for these vaccines by companies such as Moderna in Boston and CureVac in Tübingen are discussed in this paper. Despite these and other companies currently being “distracted” by COVID-19, clinical trials are ongoing for influenza, which will remain a public health issue beyond the current SAS-CoV-2 pandemic. Thus far, no RNA vaccines have been licensed by the FDA, but it is to be expected that licensing of the influenza vaccines will also receive a boost from the current focus on the “warp speed” development of vaccines against COVID-19.

Regardless of the nature of the vaccine formulation, currently, the efficacy of a vaccine for licensing purposes is established by testing only its ability to induce or increase influenza-specific antibodies. This is the only surrogate of efficiency commonly available. However, it does not guarantee clinical protection, because protection relies on cellular as well as humoral immunity, and even for the latter, it depends on how and which antibodies are assayed. As pointed out by Domnich et al. [5] in a survey of a large number of trials, methods of measuring influenza antibodies vary in different studies, and in less than one-quarter of these is any attempt also made to assess cellular immunity. This makes inter-trial comparisons of the likely efficiency of different and novel vaccines extremely difficult, and this should be a call to arms to standardize and harmonize readouts in published studies. To complicate matters further, Lewnard and Sobey [6] point out that at least some instances of unanticipated failure of even well-matched seasonal influenza vaccines may be due to the different past exposures of different segments of the population to earlier influenza infections. Much of this immune memory will also be mediated by T cells, both as CD4+ helper cells for B cell antibody production and CD8+ cytotoxic cells killing virally-infected host cells, again emphasizing the still-underappreciated importance of cellular immunity.

Much effort is currently being invested in developing vaccines that optimally stimulate such T cell immunity to overcome many of the above problems and provide protection against multiple different influenza strains by targeting antigens shared between them and not varying seasonally, as discussed in the paper by Clemens et al. [7]. This paper has already been the subject of an editorial in this journal [8] and will not be further discussed here.

Taken together, and also considering the many other papers published in the “Influenza Virus Vaccines” section and several other relevant sections of this journal (see https://www.mdpi.com/journal/vaccines/sections/Influenza_virus_vaccines), it is clear that there remain many scientific, commercial, and public health challenges to overcome before we can really feel comfortable in asserting that we have influenza anywhere near under control. The current focus on COVID-19 must not be allowed to distract us from the continuing seriousness of what is dismissed by many as “only the flu”.
